# Functional Correlations of Pathogenesis-Driven Gene Expression Signatures in Tuberculosis

**DOI:** 10.1371/journal.pone.0026938

**Published:** 2011-10-28

**Authors:** Jeroen Maertzdorf, Martin Ota, Dirk Repsilber, Hans J. Mollenkopf, January Weiner, Philip C. Hill, Stefan H. E. Kaufmann

**Affiliations:** 1 Department of Immunology, Max Planck Institute for Infection Biology, Berlin, Germany; 2 Medical Research Council Laboratories, Banjul, The Gambia; 3 Leibniz Institute for Farm Animal Biology, Genetics and Biometry, Dummersdorf, Germany; Statens Serum Institute, Denmark

## Abstract

Tuberculosis remains a major health threat and its control depends on improved measures of prevention, diagnosis and treatment. Biosignatures can play a significant role in the development of novel intervention measures against TB and blood transcriptional profiling is increasingly exploited for their rational design. Such profiles also reveal fundamental biological mechanisms associated with the pathology of the disease. We have compared whole blood gene expression in TB patients, as well as in healthy infected and uninfected individuals in a cohort in The Gambia, West Africa and validated previously identified signatures showing high similarities of expression profiles among different cohorts. In this study, we applied a unique combination of classical gene expression analysis with pathway and functional association analysis integrated with intra-individual expression correlations. These analyses were employed for identification of new disease-associated gene signatures, identifying a network of Fc gamma receptor 1 signaling with correlating transcriptional activity as hallmark of gene expression in TB. Remarkable similarities to characteristic signatures in the autoimmune disease systemic lupus erythematosus (SLE) were observed. Functional gene clusters of immunoregulatory interactions involving the JAK-STAT pathway; sensing of microbial patterns by Toll-like receptors and IFN-signaling provide detailed insights into the dysregulation of critical immune processes in TB, involving active expression of both pro-inflammatory and immunoregulatory systems. We conclude that transcriptomics (i) provides a robust system for identification and validation of biosignatures for TB and (ii) application of integrated analysis tools yields novel insights into functional networks underlying TB pathogenesis.

## Introduction

Tuberculosis (TB), caused by the intracellular bacterium *Mycobacterium tuberculosis* (*Mtb*), remains a major health threat. Frequently infection remains latent with a 10% lifetime risk of developing active TB disease. Current tools for prevention, diagnosis and treatment are inadequate, and despite the use of Bacille Calmette-Guérin (BCG) as vaccine to prevent TB, its efficacy and application practices varies worldwide [1].

Global gene transcript profiling in peripheral blood leukocytes (PBL) has evolved into a state-of-the-art measure of the status of the immune system in health and disease (reviewed in [2]). Immune cells patrol the human macroorganism and circulate through blood and lymph to relocate to different tissue sites in the body. During infection, alterations of immune processes in the infected tissue lead to changes in the transcriptional profiles of circulating immune cells. These changes in host gene expression reflect the response of the host immune system to invading pathogens.

Although gene expression profiling of PBL from *Mtb*-infected individuals has been limited, transcriptional signatures characteristic for latent infection and active disease are slowly emerging [3]–[6]. These gene signatures have already provided proof of principle for development of novel TB diagnostics by reliably discriminating *Mtb* latently infected healthy individuals from patients with active TB disease. The ultimate goal herein will be the risk assessment in latently *Mtb*-infected individuals for progression to active disease. Such disease-related signatures can also be exploited for monitoring of vaccine and drug trials by providing surrogates that predict clinical outcome at early stages of trials [7], [8].

Previous studies focusing on gene signatures indicative of TB disease versus latent infection [3], [5], [6], [9] have revealed several clusters of differentially regulated genes including genes involved in type I and II interferon (IFN) signaling, cell type-specific signatures such as increased NK cell and neutrophil activity [5], [6], involvement of *RAB33A* and *SOCS3* in T cells [9], [10], and markers of apoptosis [6]. In this study, we identify new and validate previously identified gene signatures in TB in an independent cohort from The Gambia and use a unique combination of classical gene expression, pathway, and functional association analyses integrated with intra-individual expression correlations for identification of transcriptionally regulated markers of key biological processes in TB.

## Results

### Identification of differentially expressed genes

We interrogated the transcriptomes of PBL from TB patients (TB) and healthy donors who were latently infected (LTBI) or uninfected with *Mtb* (NID) to validate previous findings in an independent cohort and to define pathognomonic markers of TB. Group-wise comparisons revealed a total of 1,661 differentially expressed genes between TB and LTBI (log2-fold change M>0.5 or M<−0.5; significance q<0.01). No obvious differences were observed between LTBI and NID when applying the same stringent cutoff values. Less stringent filtering did show significant differences between these two cohorts, although with weak differences in fold changes. Consequently, we focused on TB versus LTBI comparisons, with emphasis on functional pathways and interaction networks to gain novel insight into biological processes relevant for TB protection and pathogenesis.

Differentially expressed genes between TB and LTBI showed a highly significant enrichment of genes involved in regulation of defense and immune responses, signal transduction and activation of leukocyte populations. A full list of enriched gene sets based on gene ontology (GO) terms using the web-based tool GOrilla [11] is provided as supplemental information (Table S1).

### SPIA analysis of TB versus LTBI

**Figure 1:**
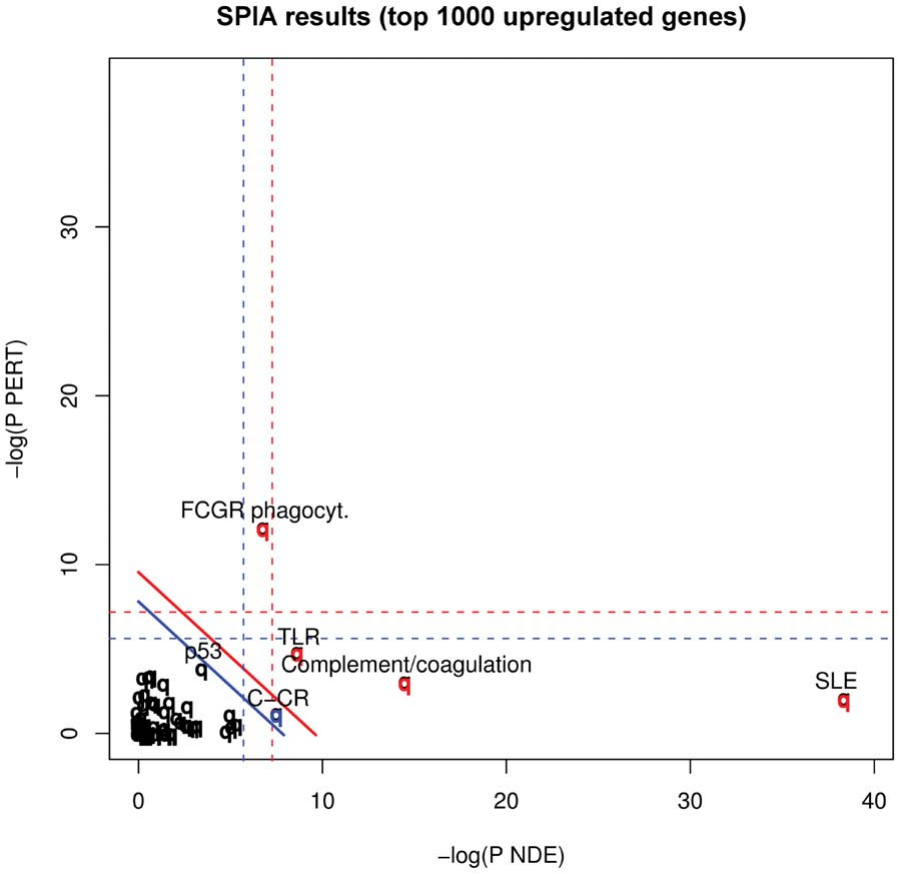
SPIA analysis on differentially expressed genes. Analysis was run on the top 1.000 ranking genes showing increased expression in TB compared to LTBI . X-axis indicates enrichment of genes; y-axis represents perturbation of genes within pathways. Pathways above the blue line are significant at 5% after FDR correction, those above the red lines at 5% after Bonferroni correction.

In an attempt to integrate differentially expressed genes into known biological pathways, we performed a signaling pathway impact (SPIA) analysis on our microarray data in this Gambian cohort. This analysis combines canonical enrichment analysis with a novel type of evidence that measures perturbation within a given pathway [12]: The x-axis depicts enrichment of gene sets based on GO descriptions; the y-axis visualizes perturbation of genes involved in 89 well-characterized KEGG (Kyoto Encyclopedia of Genes and Genomes) pathways. In this perturbation, differentially expressed genes in a higher hierarchical position within a certain pathway have higher impact on the outcome of the analysis. Simultaneously, this analysis takes into account known influences of any gene within the KEGG pathway on the expression of downstream genes. Focusing on the identification of activated pathways in TB, we performed an exploratory analysis of the top 1.000 upregulated genes (representing genes with a log2-fold change of M>0,5) in TB versus LTBI (Figure 1). This somewhat arbitrary cutoff was chosen to allow a sufficient input number of genes for the perturbation analysis, and at the same time not to weaken the gene enrichment analysis with a large input of genes showing only marginal levels of upregulation.

This SPIA analysis revealed a remarkable enrichment of genes involved in the autoimmune disease systemic lupus erythematosus (SLE). This enrichment of differentially expressed genes is mainly dominated by the involvement of increased histone expression in TB. Other KEGG pathways with significantly enriched gene sets in The Gambian cohort involved components of the complement and coagulation cascades, TLR signaling and, to a lesser degree, cytokines and their homologous receptors. Differential expressions between TB and LTBI in The Gambian cohort for all genes in these pathways are depicted in Table S2.

**Figure 2:**
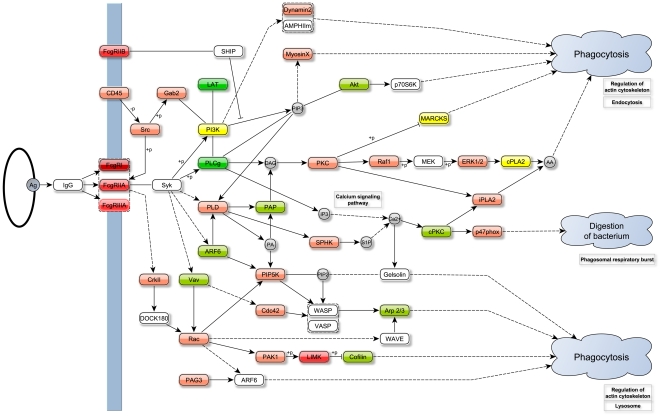
KEGG pathway of Fc gamma receptor-mediated phagocytosis. Items colored red and green indicate respectively up- and down-regulated genes in TB as compared to LTBI (light-to-dark colors indicate medium to high differential expression); yellow indicates clusters of genes including both up- and down-regulated genes. Figure was recreated and modified based on KEGG pathway hsa0466 (http://www.genome.jp/kegg/pathway/hsa/hsa04666.html).

Last but not least, analysis of these data revealed a significant perturbation within the Fc gamma receptor-mediated phagocytosis pathway. Figure 2 illustrates the complete KEGG pathway for Fc gamma receptor-mediated phagocytosis with coloring, permitting visualization of the differential expression of genes involved.

### Correlations in gene expression

**Figure 3:**
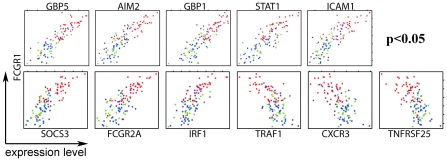
Correlations of gene expression in cohort subjects. Genes showing a highly significant correlation of expression with *FCGR1* in all subjects in the Gambian cohort. Red dots indicate expression levels in TB patients, blue in LTBI and green of NID donors. X and Y axes represent normalized expression levels of individual genes.

Biological variation between individuals within each group could obscure expression differences of relevant genes. We therefore analyzed gene expression levels and correlations between genes in single individuals, irrespective of fold changes and significance values in the group-wise comparisons. We chose *FCGR1* as starting point for correlation analyses as one of the most differentially expressed genes in both African cohorts (The Gambia and South Africa), which also showed strongest discriminatory power for expression profiles of TB patients and LTBI donors (data not shown). Figure 3 illustrates expression correlations between *FCGR1* and several highly significantly correlated genes. A list of the top 100 best correlated genes, both positively and negatively, is provided as supplementary material (Table S3). In contrast to group-wise comparisons, these plots demonstrate correlations in expression levels between genes in individuals from each group. While expression levels of these exemplary genes discriminate between TB and LTBI, they also indicate biological variation leading to a degree of overlap between groups. In contrast, expression levels between LTBI and NID were not significantly different, neither by group-wise comparisons nor by gene correlation analysis. Intriguingly, in two individuals of the TB group, gene expression signatures resembled profiles characteristic for the LTBI and NID groups. It remains to be established why these individuals displayed a signature similar to healthy individuals, since clinical diagnosis by consecutive sputum smear-positive tests unequivocally grouped these individuals in the active TB group.

### Functional correlation of differentially expressed genes

**Figure 4:**
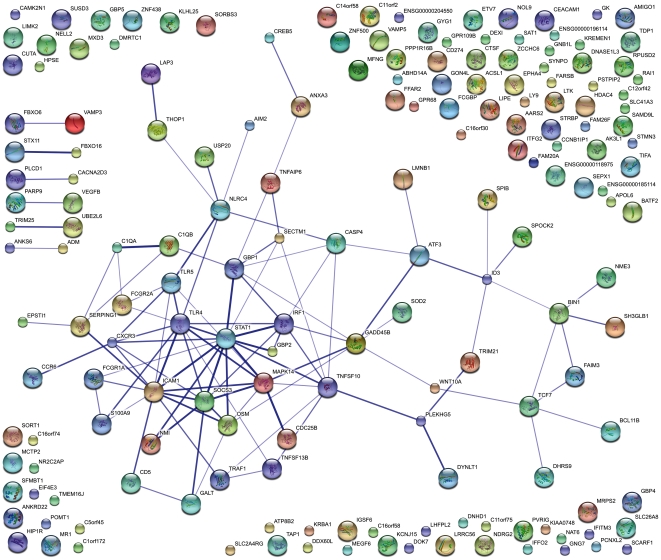
Functional association network of FCGR1-correlated genes. The STRING functional association network was generated using the top 100 positively correlated, plus the top 100 negatively correlated genes. Shown here is the confidence view.

Correlations of differentially expressed genes between TB and LTBI, at least in part, may reflect differences in cell type composition between individuals in either group. In an attempt to elucidate the functional relevance of this biological variation, we analyzed 200 genes that showed highest expression correlation with *FCGR1* (top 100 highest positive and top 100 highest negative correlations). Functional association analysis of these genes using the manually curated STRING database [13] identified a network of 71 gene products (Figure 4); a high resolution version is also provided as supplementary material (Figure S1) with highly interacting gene products centered around the JAK-STAT pathway (*STAT1*, *SOCS3*; previously also shown in the South African cohort [9]), *ICAM1*, TLRs, Fc gamma receptors, complement factors and IFN-response genes. The full list of these interacting genes with their respective differential expression between TB and LTBI is provided as supplementary material (Table S4; sheet 1). Some of these correlating genes (*FBXO16*, *TNFRSF10* and *TNFRSF17*) expressed poor q-values in the group-wise comparisons, underlining the power of expression correlations and the potential risk of dismissing relevant genes due to biological variation within single groups and cohorts. In a final analysis we assessed functional relations of genes within this network. To this end, a functional annotation analysis of the 71 genes within this functional interaction network was performed using the online tool DAVID [14]. Results of this functional annotation demonstrated profound associations within this network of genes involved in immune inflammatory responses, apoptosis, responses to bacterial molecular patterns and the NF-kappa B cascade (see Table S4; sheet 2).

### Validation of previously identified expression profiles

Previous studies by our group [3], [6], identified gene profiles which discriminated between TB and LTBI, including a group of interferon (IFN)-inducible genes, which were markedly upregulated in TB patients. A recent publication by another group [5] described a whole-blood transcription signature in TB patients dominated by a neutrophil-driven IFN-inducible gene profile, consisting of both IFN-γ and type I IFN-αβ signaling. For validation purposes, we compared these signatures (cohort UK), with expression profiles from cohorts in South Africa (SUN; published in [6]) and The Gambia (MRC; described here).

**Figure 5:**
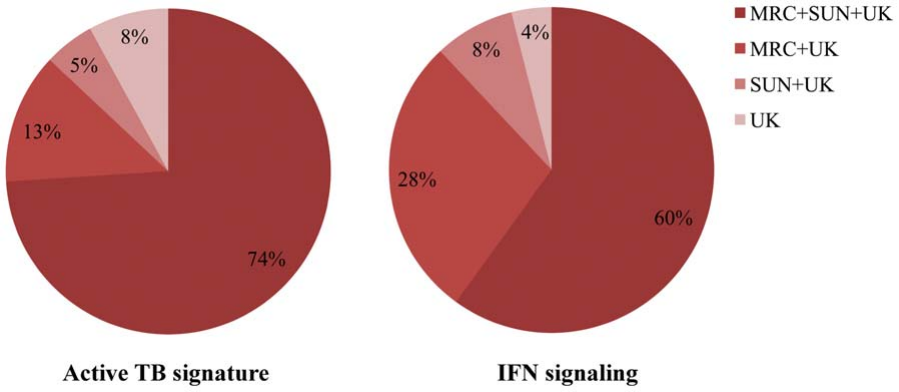
Comparison of differential expression in three independent cohorts. Comparison of differential gene expression between TB and LTBI in cohorts in South Africa (SUN) and The Gambia (MRC) with gene profiles in a cohort in the United Kingdom (UK) identified by Berry, et al. (Berry 2010). Significance of differential expression at corrected p-value level of q<0.05.

Figure 5 depicts comparisons between the three different cohorts, showing statistically significant differences in gene expression between TB and LTBI. As many as 92% of the genes within the TB signature and 96% of the IFN-inducible gene signature identified in the UK cohort [5] did show significant (at q<0.05) differential expression between TB and LTBI in either one or both of the African cohorts. A full list of matching gene IDs and expression data from both gene profiles is provided as supplementary material (Table S5). Thus transcriptomics allows robust correlation of distinct clusters of differentially expressed genes among cohorts from different geographical locations and ethnic origins.

## Discussion

Current diagnostics for TB are inaccurate and slow, underlining the urge for better and swifter measures [15]. New strategies should aim at developing a point-of-care test for TB [16]. In addition to tests that rely on identifying *Mtb* or its products, host genetic profiles provide a valuable source for the development of new diagnostic tools. Such host profiles can also provide in-depth insights into functional mechanisms of protection and pathogenesis in TB. Rational design of a host biosignature for TB should include elucidation of the relationship of differentially expressed genes within functional clusters and requires validation of candidate biomarker genes and signatures [7], [17]. Our analysis reveals a group of differentially expressed genes which (i) perform relevant biological functions in TB and (ii) are shared in different populations and identified by different analytical systems. Hence, the identification of new TB-related gene expression profiles and validation of previously identified signatures herein, represents a valuable contribution towards pathogenesis-based transcriptomic diagnosis of TB.

During pulmonary infection with *Mtb*, different immune processes are induced in the affected tissue and are accompanied by infiltration of immune cells. These alterations are succeeded by changes in the gene expression profile of re-circulating immune cells in the peripheral blood. Due to their easy accessibility, peripheral blood leukocytes have become the preferred surrogate targets for analyzing disease-related biomarkers in TB patients and LTBI due to their easy accessibility. Previous studies on gene transcription profiling in peripheral blood cells of TB patients have identified signatures of differentially expressed genes [3]–[6], [9]. Our demonstration of highly similar signatures and the identification of new profiles represents a major step forward towards true biosignatures for TB. Validation of several of these signatures across cohorts from different geographical locations and ethnical background, identified by independent research groups using different platforms and analytical methods, demonstrates robustness of these biomarker signatures.

Our analysis of expression profiles focused on functional pathways and interaction networks to gain in-depth insights into biological processes underlying protection and pathogenesis in TB. Signaling pathway impact analysis (SPIA) of the top 1,000 most differentially expressed genes between TB and LTBI revealed an unexpected enrichment of genes that are involved in SLE. This systemic autoimmune disease is characterized by deposition of immune complexes (IC) and a gene expression profile of elevated type I IFN signaling [18], [19] similar to what was observed in TB (this study and [5], [6]). The causes of this elevated type I IFN expression in SLE are likely deposition of ICs triggering IFNα production by plasmacytoid dendritic cells through Fc gamma receptors [19]. Elevated levels of IC accompanied by defective IC solubilization has also been documented in serum of TB patients [20] and may account, at least in part, for the similarities in gene signatures observed in both diseases. Reduced solubilization of IC also has a well-known pathological manifestation in leprosy, notably during malignant stages [21], which is caused by the mycobacterial pathogen *M. leprae*. The observed increased expression of TLRs as described here, as key molecules in type I IFN signaling, likely further contributes to the IFN-inducible gene signature observed in both SLE and TB.

In the cohort from The Gambia described herein, the gene *FCGR1* evolved as one of the strongest differentially expressed genes between TB and LTBI, with the highest discriminative power between the two groups. This is in accordance with our previous observations in other cohorts, namely from Europe and South Africa [3], [6]. Upregulated expression of *FCGR1* in TB patients followed by alterations in downstream pathways are a clear hallmark of active TB disease, as identified through SPIA analysis (Figure 2) and analysis of differential gene expression patterns involved in the KEGG pathway of Fc gamma receptor-mediated phagocytosis (Figure 3).

A confounding aspect when studying gene expression profiles in many diseases including TB is the inherent biological variation between individuals within study populations, which is most pronounced in the active TB group. An intriguing example for this variation is the identification of two patients in the cohort described herein, which displayed an expression signature that closely resembled that of healthy individuals. Expression levels of correlating genes in figure 4 and a heatmap provided as supplementary material (Figure S2) illustrate these “outliers”. Although we cannot formally exclude that the two outlier TB patients in this study were diseased from non-tuberculous mycobacteria, from a clinical perspective this is unlikely in a cohort of HIV-negative individuals, where non-tuberculous disease cases are uncommon. Previous studies have speculated about outliers within the LTBI group with a signature indicative of active TB as sub-clinical active cases [5] or individuals progressing to active TB before the onset of clinical symptoms [4]. Our findings revealing outliers in the active TB group with a signature characteristic of the healthy groups emphasize the occurrence of outliers as a general phenomenon in all groups and hence caution is advised on speculating about their prognostic value before definite proof has been obtained. Reliable interpretation will depend on data from longitudinal follow-up studies such as LTBI who progress into active TB disease as well as of cured TB patients with or without relapses.

When analyzing group-wise comparisons, variations amongst individual group members can obscure disease-related markers and profiles, which prompted us to embark on further analyses of correlations in gene expression among individuals of the different groups. Taking *FCGR1* as the starting point for these analyses, a large group of genes was identified that revealed both positive and negative correlations with *FCGR1* expression. In part, these correlations also reflect differences in cell type compositions between subjects, which inherently contribute to inter-individual variation. Biological association analysis of 200 genes with the highest expression correlation (both positive and negative) revealed a network of highly interrelated gene products. Three genes within this functional network had a very poor significance value (q>0.1) in a group-wise comparison between TB and LTBI, underlining the power of analyzing gene expression correlations and the potential risk of dismissing relevant genes due to biological variation within groups. Functional annotation analysis of the genes in this network indicated strong association with immune inflammatory responses, apoptosis, responses to bacterial molecular patterns and the NF-kappa B cascade. Central to this network are pro-inflammatory regulators with interactions involving the JAK-STAT pathway – *STAT1*, *SOCS3*; previously also shown in the South African cohort [9] – *ICAM1*, TLRs, Fc gamma receptors and complement factors and IFN-response genes.

Elevated expression in TB patients of IFN-related genes, TLRs and genes involved in the JAK-STAT signaling pathway (*STAT1*, *OSM* and *NMI*) at first sight seems contradictory to the increased expression of *SOCS3*, known to inhibit signal transduction emanating from cytokine and TLR stimulation through the JAK-STAT pathway [22], [23]. In a similar way, our data indicate exacerbated expression of complement factors, despite increased expression of the complement cascade silencing molecule *SERPING1* [24]. These findings indicate the activation of inhibitory feedback loops in presence of respective activating signaling cascades. Future studies are needed to clarify whether these feedback loops effectively participate in fine-tuning of effector cascades or are activated in vein in TB.

Blood transcript profiling is emerging as a common tool for understanding the status of the human immune response during systemic and infectious diseases [2]. Here we describe the identification of new pathogenesis-driven gene expression signatures and validation of previously identified gene profiles from other cohorts in TB. We have focused on functional pathways and interaction networks to gain in-depth insights into biological processes relevant to protection and pathogenesis in TB which can be harnessed for relevant biomarker signatures. Altered expression of genes in these biological pathways provides a deeper insight into the dysregulation of key regulatory immune processes in the progression from LTBI to TB disease.

Together these results provide a better understanding of the immune pathology in TB, while combined biosignatures may be employed into the development of a pathogenesis-based point-of-care test.

## Methods

### Ethics statement

The study presented here was approved by the Gambia Government/Medical Research Council Joint Ethics Committee in The Gambia and by the Ethical committee 1 on Campus Charite – Mitte in Berlin, Germany. Written informed consent was obtained from all study participants.

### Subject enrolment and sample collection

In this study, a total of 46 sputum smear and chest x-ray positive TB patients (TB), 25 latently infected healthy donors (LTBI, TB case contacts with Mantoux test induration > = 10 mm) and 37 uninfected donors (NID, Mantoux test induration  = 0 mm) were included from a cohort recruited at the Medical Research Council Laboratories (MRC) in Banjul, The Gambia. All subjects were HIV^–^ and samples from TB patients were taken prior to chemotherapy. General characteristics of the study subjects in this cohort are given in Table 1. From every donor, 2.5 ml of peripheral whole blood was collected in PAXgene tubes (PreAnalytix) and stored at −80°C prior to processing.

**Table 1:** Characteristics of subjects in the cohort from The Gambia.

	TB	LTBI	NID
Number of subjects	46	25	37
Females/Males	21/25	15/10	21/16
Mean age (range)	30 (16–53)	29 (16–54)	30 (16–54)

### RNA extraction and microarray procedure

Extraction and processing of RNA were performed as described previously [6]. Briefly, RNA was extracted using the PAXgene Blood RNA Kit (PreAnalytix) and labeled with the Fluorescent Linear Amplification Kit (Agilent Technologies) according to manufacturer's instructions. Quantity and labeling efficiency were verified before hybridization of the samples to whole-genome 4×44 k human expression arrays (Agilent) and scanned at 5 µm using an Agilent scanner.

### Data analysis

Analysis of the scanned images was performed with Feature Extraction software (version 6.1.1, Agilent Technologies). Data analysis was performed using the R package. Data were log-transformed and differentially expressed genes were identified based on log2 fold changes (M-values) in average gene expression with a q value <0.01 (q equals the p value corrected for multiple testing). Microarray data are in compliance to MIAME guidelines and have been deposited in the GEO database under accession GSE28623.

### Bio-statistical analysis

Enrichment analysis of differentially expressed genes based on Gene Ontology terms was performed using the web-based tool GOrilla (http://cbl-gorilla.cs.technion.ac.il) [11]. Signaling pathway impact analysis (SPIA), combining classic gene enrichment and pathway perturbation was performed using an implemented R package [12]. Functional association studies were performed using the manually curated STRING database (online interface available under http://string-db.org) [13] and functional annotation of genes in networks was performed using the web-based tool DAVID (http://david.abcc.ncifcrf.gov) [14].

## Supporting Information

Figure S1 **Functional association network of FCGR1 correlating genes.** STRING network showing functional associations between FCGR1-correlated genes (high-resolution version of Figure 4).
(PNG)

Figure S2 **Heatmap of differentially expressed genes.** Indicated are the 50 most differentially expressed genes between TB patients and healthy donors (TST_Neg and TST_Pos). TB patients with expression patterns similar to healthy individuals are outlined. Normalized expression levels are colored blue to red (low to high expression resp.).
(PNG)

Table S1 **Gene Ontology enrichment of differentially expressed genes.** Enrichment analysis of genes differentially expressed between TB and LTBI was performed using the DAVID bioinformatics tool (Huang 2009).
(XLS)

Table S2 **KEGG pathway genes identified by SPIA analysis.** Full lists of genes in the pathways identified in the SPIA analysis and their differential expression between TB and LTBI within the cohort from The Gambia. A few gene names derived from the KEGG pathways could not be identified within the expression data from our microarray platform. Genes with significant differential expression between TB and LTBI are indicated in black; genes with significance values of q>0.05 are indicated in grey.
(XLS)

Table S3 **Genes with expression correlation to FCGR1.** List of the top 100 genes showing strongest positive and negative correlation with expression of FCGR1 (sheet 1 indicates top 100 positively correlated; sheet 2 top 100 negatively correlated genes).
(XLS)

Table S4 **Differential expression and annotation of genes in STRING network.** Genes within the functional association network depicted in Figure 5 and their differential expression between TB and LTBI, and correlation with FCGR1. Sheet 2 shows functional annotation of these genes as analyzed by the online DAVID tool.
(XLS)

Table S5 **Genes within the TB and IFN-inducible signatures.** Lists of genes within the TB signature and interferon signaling, described for the UK cohort by Berry et al [5] that match annotations of transcripts in cohorts from The Gambia (MRC) and South African (SUN). The original 393 transcript TB signature identified in the UK cohort represented 307 unique gene IDs, of which 288 matched with annotations in the expression data from the Gambian and South African cohorts. Indicated are fold changes on a log2 scale (M) and statistical significance (q) of differential expression between TB and LTBI. Positive M values indicate increased expression in TB compared to LTBI.
(XLS)

## References

[1] Zwerling A, Behr MA, Verma A, Brewer TF, Menzies D (2011). The BCG world atlas: A database of global BCG vaccination policies and practices.. PloS Medicine.

[2] Chaussabel D, Pascual V, Banchereau J (2010). Assessing the human immune system through blood transcriptomics.. BMC Biology.

[3] Jacobsen M, Repsilber D, Gutschmidt A, Neher A, Feldmann K (2007). Candidate biomarkers for discrimination between infection and disease caused by Mycobacterium tuberculosis.. J Mol Med.

[4] Mistry R, Cliff JM, Clayton CL, Beyers N, Mohamed YS (2007). Gene-expression patterns in whole blood identify subjects at risk for recurrent tuberculosis.. J Infect Dis.

[5] Berry MP, Graham CM, McNab FW, Xu Z, Bloch SA (2010). An interferon-inducible neutrophil-driven blood transcriptional signature in human tuberculosis.. Nature.

[6] Maertzdorf J, Repsilber D, Parida SK, Stanley K, Roberts T (2011). Human gene expression profiles of susceptibility and resistance in tuberculosis.. Genes Immun.

[7] Parida SK, Kaufmann SH (2010). The quest for biomarkers in tuberculosis.. Drug Discov Today.

[8] Zárate-Bladés CR, Silva CL, Passos GA (2011). The Impact of Transcriptomics on the Fight against Tuberculosis: Focus on Biomarkers, BCG Vaccination, and Immunotherapy.. Clin Dev Immunol.

[9] Jacobsen M, Repsilber D, Kleinsteuber K, Gutschmidt A, Schommer-Leitner A (2010). Suppressor of cytokine signaling (SOCS)-3 is affected in T cells from TB patients.. Clin Microbiol Inf.

[10] Jacobsen M, Repsilber D, Gutschmidt A, Neher A, Feldmann K (2005). Ras-associated small GTPase 33A, a novel T cell factor, is down-regulated in patients with Tuberculosis.. J Inf Dis.

[11] Eden E, Navon R, Steinfeld I, Lipson D, Yakhini Z (2009). GOrilla: A Tool For Discovery And Visualization of Enriched GO Terms in Ranked Gene Lists.. BMC Bioinformatics.

[12] Tarca AL, Draghici S, Khatri P, Hassan SS, Mittal P (2009). A novel signaling pathway impact analysis.. Bioinformatics.

[13] Szklarczyk D, Franceschini A, Kuhn M, Simonovic M, Roth A (2011). The STRING database in 2011: functional interaction networks of proteins, globally integrated and scored. Nucleic Acids Res 39(Database issue):D561-8.. Epub 2010 Nov.

[14] Huang da W, Sherman BT, Lempicki RA (2009). Systematic and integrative analysis of large gene lists using DAVID bioinformatics resources.. Nat Protoc.

[15] Wallis RS, Pai M, Menzies D, Doherty TM, Walzl G (2010). Biomarkers and diagnostics for tuberculosis: progress, needs, and translation into practice.. Lancet.

[16] McNerney R, Daley P (2011). Towards a point-of-care test for active tuberculosis: obstacles and ooportunities.. Nat Rev Microbiol.

[17] Doherty M, Wallis RS, Zumla A, WHO-Tropical Disease Research/European Commission joint expert consultation group (2009). Biomarkers for tuberculosis disease status and diagnosis.. Curr Opin Pulm Med.

[18] Obermoser G, Pascual V (2010). The interferon-alpha signature of systemic lupus erythematosus.. Lupus.

[19] Kyogoku C, Tsuchiya N (2007). A compass that points to lupus: genetic studies on type I interferon pathway.. Genes Immun.

[20] Senbagavalli P, Geetha ST, Venkatesan P, Ramanathan VD (2009). Defective solubilization of immune complexes and activation of the complement system in patients with pulmonary tuberculosis.. J Clin Immunol.

[21] Ramanathan VD, Tyagi P, Ramanathan U, Katoch K, Sengupta U (1991). Persistent reduced solubilization of immune complexes in lepromatous leprosy patients with reactions.. Int J Lepr Other Mycobact Dis.

[22] Alexander WS, Hilton DJ (2004). The role of suppressors of cytokine signaling (socs) proteins in regulation of the immune response.. Annu Rev Immunol.

[23] Dimitriou ID, Clemenza L, Scotter AJ, Chen G, Guerra FM (2008). Putting out the fire: Coordinated suppression of the innate and adaptive immune systems by socs1 and socs3 proteins.. Immunol Rev.

[24] Davis AE (2004). Biological effects of C1 inhibitor.. Drug News Perspect.

