# Supplementary figures and images for: Functional Correlations of Pathogenesis-Driven Gene Expression Signatures in Tuberculosis
Source: PLoS One. 2011 Oct 28;6(10):e26938. doi: 10.1371/journal.pone.0026938 (PMC3203931; doi:10.1371/journal.pone.0026938)

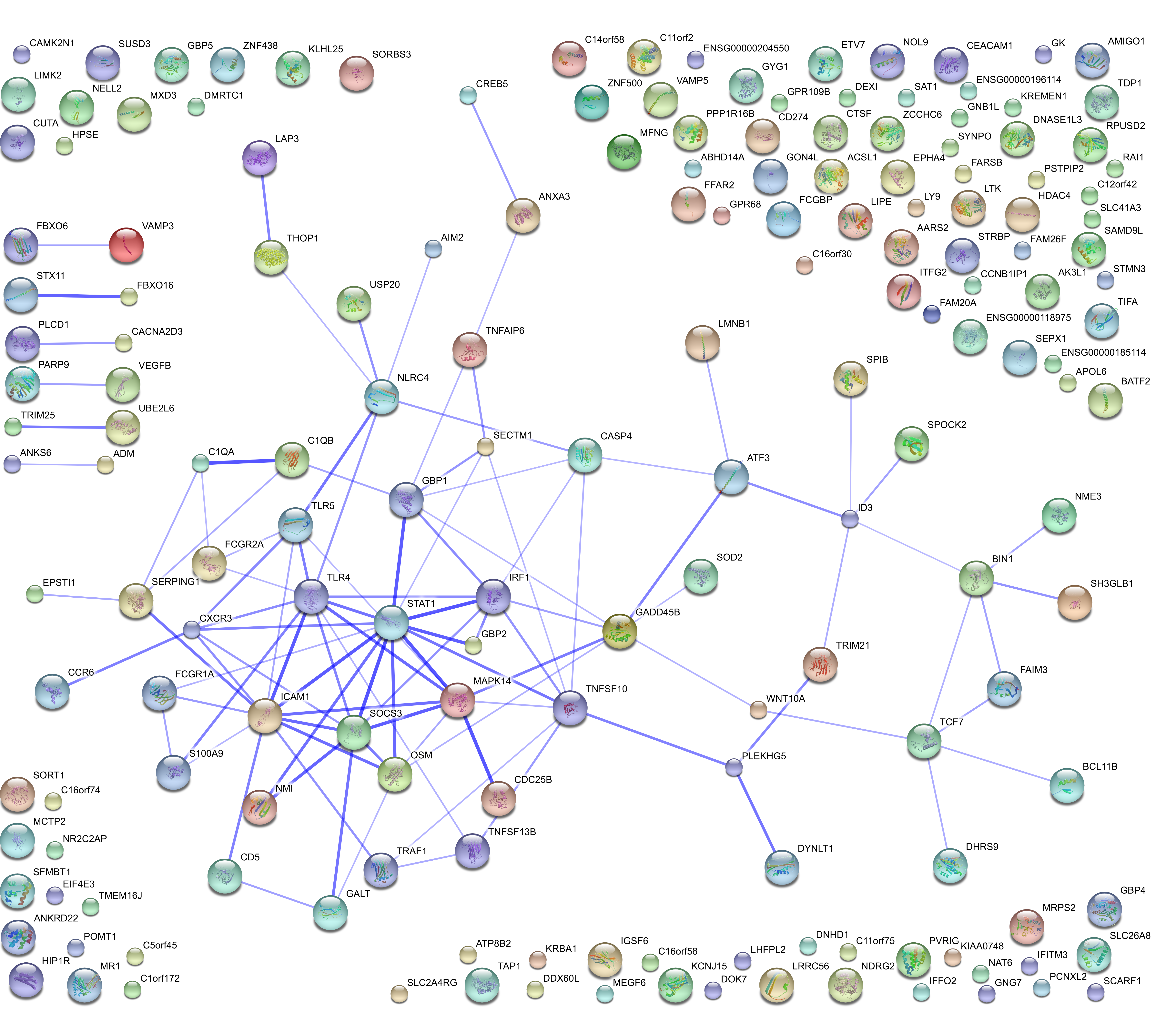

Supplement: Figure S1 — Functional association network of FCGR1 correlating genes. STRING network showing functional associations between FCGR1-correlated genes (high-resolution version of Figure 4). (PNG) [file pone.0026938.s001.png]

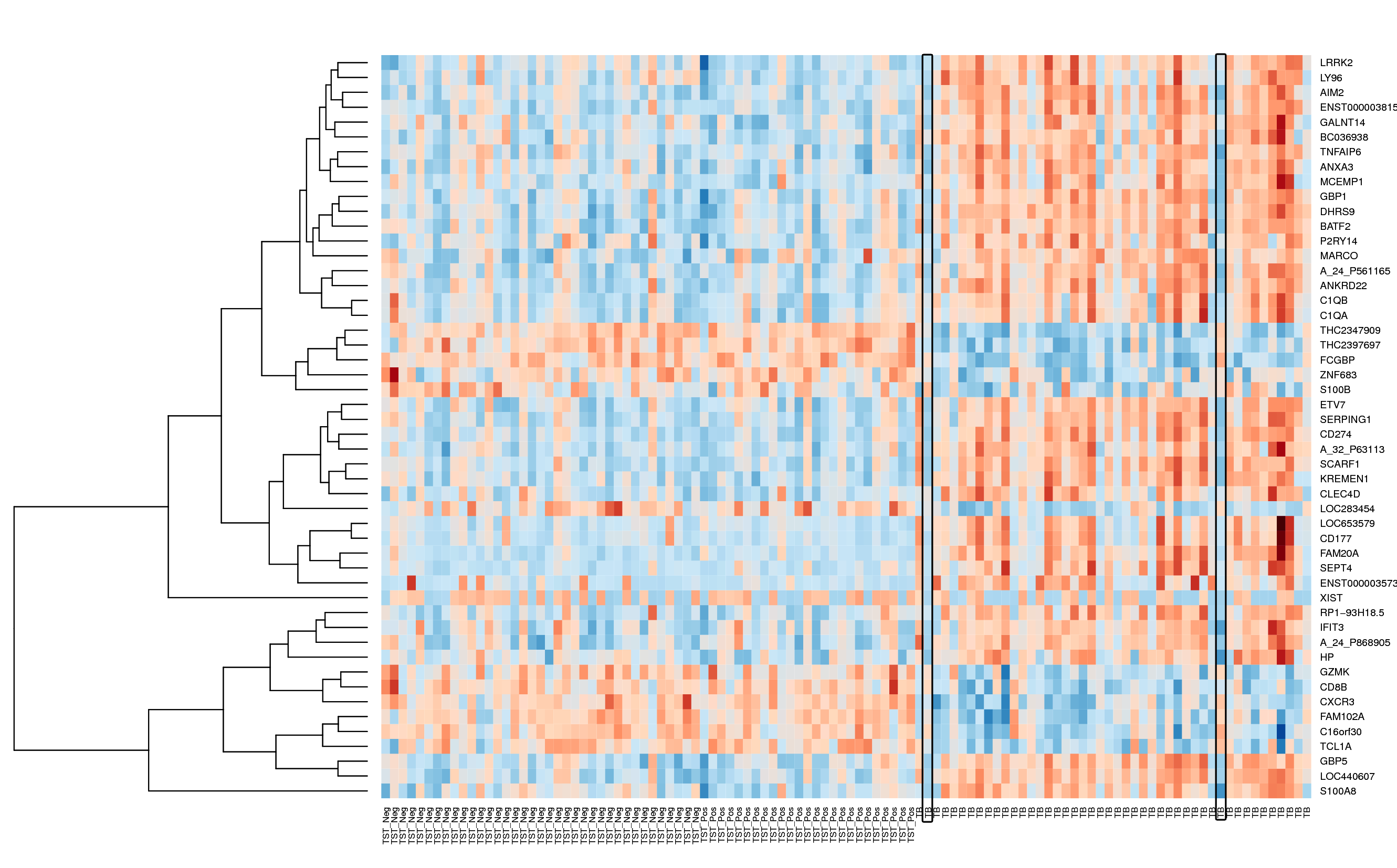

Supplement: Figure S2 — Heatmap of differentially expressed genes. Indicated are the 50 most differentially expressed genes between TB patients and healthy donors (TST_Neg and TST_Pos). TB patients with expression patterns similar to healthy individuals are outlined. Normalized expression levels are colored blue to red (low to high expression resp.). (PNG) [file pone.0026938.s002.png]
